# Mesenchymal Stem Cell-Derived Extracellular Vesicles: A Potential Therapeutic Strategy for Acute Kidney Injury

**DOI:** 10.3389/fimmu.2021.684496

**Published:** 2021-06-03

**Authors:** Jia-Kun Li, Cheng Yang, Ying Su, Jing-Chao Luo, Ming-Hao Luo, Dan-Lei Huang, Guo-Wei Tu, Zhe Luo

**Affiliations:** ^1^ Department of Critical Care Medicine, Zhongshan Hospital, Fudan University, Shanghai, China; ^2^ Department of Urology, Zhongshan Hospital, Fudan University, Shanghai, China; ^3^ Shanghai Medical College, Fudan University, Shanghai, China; ^4^ Department of Critical Care Medicine, Xiamen Branch, Zhongshan Hospital, Fudan University, Xiamen, China

**Keywords:** acute kidney injury, mesenchymal stem cell, extracellular vesicle, cytokine, tubular epithelial cell

## Abstract

Acute kidney injury (AKI) is a common and potential life-threatening disease in patients admitted to hospital, affecting 10%–15% of all hospitalizations and around 50% of patients in the intensive care unit. Severe, recurrent, and uncontrolled AKI may progress to chronic kidney disease or end-stage renal disease. AKI thus requires more efficient, specific therapies, rather than just supportive therapy. Mesenchymal stem cells (MSCs) are considered to be promising cells for cellular therapy because of their ease of harvesting, low immunogenicity, and ability to expand *in vitro*. Recent research indicated that the main therapeutic effects of MSCs were mediated by MSC-derived extracellular vesicles (MSC-EVs). Furthermore, compared with MSCs, MSC-EVs have lower immunogenicity, easier storage, no tumorigenesis, and the potential to be artificially modified. We reviewed the therapeutic mechanism of MSCs and MSC-EVs in AKI, and considered recent research on how to improve the efficacy of MSC-EVs in AKI. We also summarized and analyzed the potential and limitations of EVs for the treatment of AKI to provide ideas for future clinical trials and the clinical application of MSC-EVs in AKI.

## Introduction

Acute kidney injury (AKI) is a common and sometimes life-threatening disease in patients admitted to hospital, affecting 10%–15% of all hospitalizations and around 50% of patients in the intensive care unit (1). AKI is mainly caused by ischemia reperfusion injury (IRI), medication toxicity, and sepsis (1). Severe, recurrent, and uncontrolled AKI progresses to chronic kidney disease (CKD) or end-stage renal disease (2, 3). Treatments are currently limited to dialysis and kidney transplantation; however, these are restricted by the shortage of donor organs and high costs (4, 5), and there is thus a need to develop new and effective ways of treating AKI. The common pathological features of AKI include renal tubular epithelial cell (TEC) damage (6). TECs are injured as a result of an excessive inflammatory response, and undergo apoptosis *via* Bax/Bcl pathway activation (7, 8). In addition, the mechanistic target of rapamycin/mitogen-activated protein kinase (MAPK) signaling is consistently activated in TECs and the mitochondria are damaged, leading to maladaptive repair of the injured TECs, interstitial fibrosis, and the progression of AKI into CKD (9–12). Meanwhile, expression changes in various cytokines can be detected in AKI, including vascular endothelial growth factor (VEGF), hepatocyte growth factor (HGF), and insulin-like growth factor-1 (IGF-1) (13, 14). These mechanisms involving TECs are regarded as potential therapeutic targets in AKI.

Different types of stem cells have recently been transplanted to prevent kidney damage. Among these, mesenchymal stem cells (MSCs) are considered to be one of the most promising types of cells for cellular therapy because of their ease of harvesting, low immunogenicity, and ability to be stored and expanded *in vitro* (15, 16). Notably, numerous preclinical and clinical studies have confirmed the potential role of MSCs in kidney protection and repair (17–22). However, the mechanisms by which MSCs exert their therapeutic effects remain controversial. Researchers previously believed that MSCs replaced injured cells through differentiation after introduction into the body, but this view was challenged by the fact that MSCs disappeared from the injured kidney and other organs within 72 h after administration, suggesting that differentiation and replacement of damaged cells by stem cells is probably a rare and late event in AKI *in vivo* (22, 23).

Several studies have also demonstrated that MSCs depend on complex and powerful endocrine and paracrine functions, and secrete extracellular vesicles to promote the recovery of renal function in AKI (24, 25). Secretion of growth factors, regulation of the inflammatory response, promotion of mitosis and cell proliferation, anti-apoptosis and anti-inflammatory effects, the reduction of fibrosis, and the promotion of angiogenesis have been reported in multiple studies (17). However, the main therapeutic effects of MSCs appear to be mediated by MSC-EVs, rather than by the MSCs themselves (26). Compared with MSCs, MSC-EVs have lower immunogenicity, easier storage, no tumorigenesis, and the ability to be artificially modified (24, 27–29). Increasing numbers of researchers have accordingly recognized the curative potential of MSC-EVs for AKI, and extensive preclinical research has proven their effectiveness and safety in AKI (30).

In this paper, we review the possible therapeutic mechanisms of MSCs and MSC-EVs in AKI, and consider recent research aimed at improving the therapeutic efficacy of MSC-EVs in AKI. Finally, we summarize and analyze the potential and limitations of EVs for the treatment of AKI, to provide ideas for future clinical trials and clinical applications of MSC-EV-based therapy.

## Mechanisms of MSC Therapy in AKI

MSCs are the most widely used cells for AKI treatment and allograft protocols because they can be obtained from bone marrow and expanded in culture (17). MSCs originate from the mesoderm and have the ability to differentiate into mesenchymal and non-mesenchymal cell lines, including bone and cartilage (17).

Although MSCs are mainly obtained from bone marrow, they can also be isolated from other tissues such as liver, muscle, adipose tissue, and cord blood. Such cells are distinguished by their adherent growth in culture, expression of CD90, CD73, and CD105, and lack of expression of CD34, CD45, CD19, CD11a, and human leukocyte antigen-DR (18).

Previous studies indicated that the therapeutic effect of MSCs was largely dependent on their homing ability to injured organs (31). MSCs rely on their homing ability to localize in damaged tissues. In addition to their anti-inflammatory and vascular-support effects, the homing ability of MSCs supplements their paracrine function, and is involved in protecting microvessel density (32). Transplanted MSCs detect signals from injured kidney cells and are chemoattracted to the damaged site (33). During AKI, endothelial cells express high levels of tumor necrosis factor (TNF) and interleukins (ILs), which can up-regulate the β subunit of very late antigen-4 and vascular adhesion molecule 1 to mediate the effect of bone marrow MSCs on endothelial cell adhesion (33). CXC motif chemokine receptor 4 (CXCR4) is a specific receptor for chemokine stromal cell-derived factor-1 (CXCL12), and CXCR4 cells are responsible for the renal repair function in AKI (34). Location of the damaged tissue by MSCs can be mediated by stromal cell-derived factor-1, which is robustly up-regulated during AKI and mobilizes CXCR4 cell homing to the injured kidney tissue (35).

MSCs can exert significant therapeutic effects in terms of repairing the injured kidney and improving renal function after AKI, and are distributed to the spleen, lung, lymph nodes, and kidney following intravenous administration (36). However, intravenously administered MSCs disappear within 72 h, although some MSCs can remain around the glomerulus and perirenal capillaries following renal artery injection (37), suggesting that MSCs are unlikely to treat AKI through differentiation and the replacement of damaged cells. Meanwhile, MSCs located in the lung were also shown to affect AKI through a secretory function (38), and the hypothesis that the therapeutic effects of MSCs are mainly caused by a secretory mechanism appears to be more credible. Indeed, MSCs are involved in immune regulation, anti-inflammation, anti-apoptosis, and angiogenesis promotion in the local microenvironment in injured tissue, unrelated to their differentiation ability (39). Animal models of AKI are accompanied by significant changes in cytokines, including VEGF, HGF, epidermal growth factor (EGF), IGF-1, and transforming growth factor-β (TGF-β), which participate in endothelial cell apoptosis during AKI (40). These growth factors are also widely recognized as essential components in cell regeneration and kidney repair (41). MSCs secrete molecules directly or secrete EVs that carry molecules such as VEGF, HGF, IGF-1, IL-10, fibroblast growth factor (FGF), and TGF-α, and down-regulate the inducibility of related proinflammatory molecules (such as IL-1b and TNF-α), thus, having anti-inflammatory and anti-apoptosis effects and promoting kidney repair (42). Expression levels of related cytokines have been shown to be up-regulated during the treatment of AKI with MSCs, while kidney tissue inflammation was reduced, indicating the relationship between cytokines and kidney repair in MSC-based therapy; however, more studies are needed to identify the specific mechanism involved (43).

## MSC-EVs Mediate the Therapeutic Effects of MSCs in AKI

**Figure 1:**
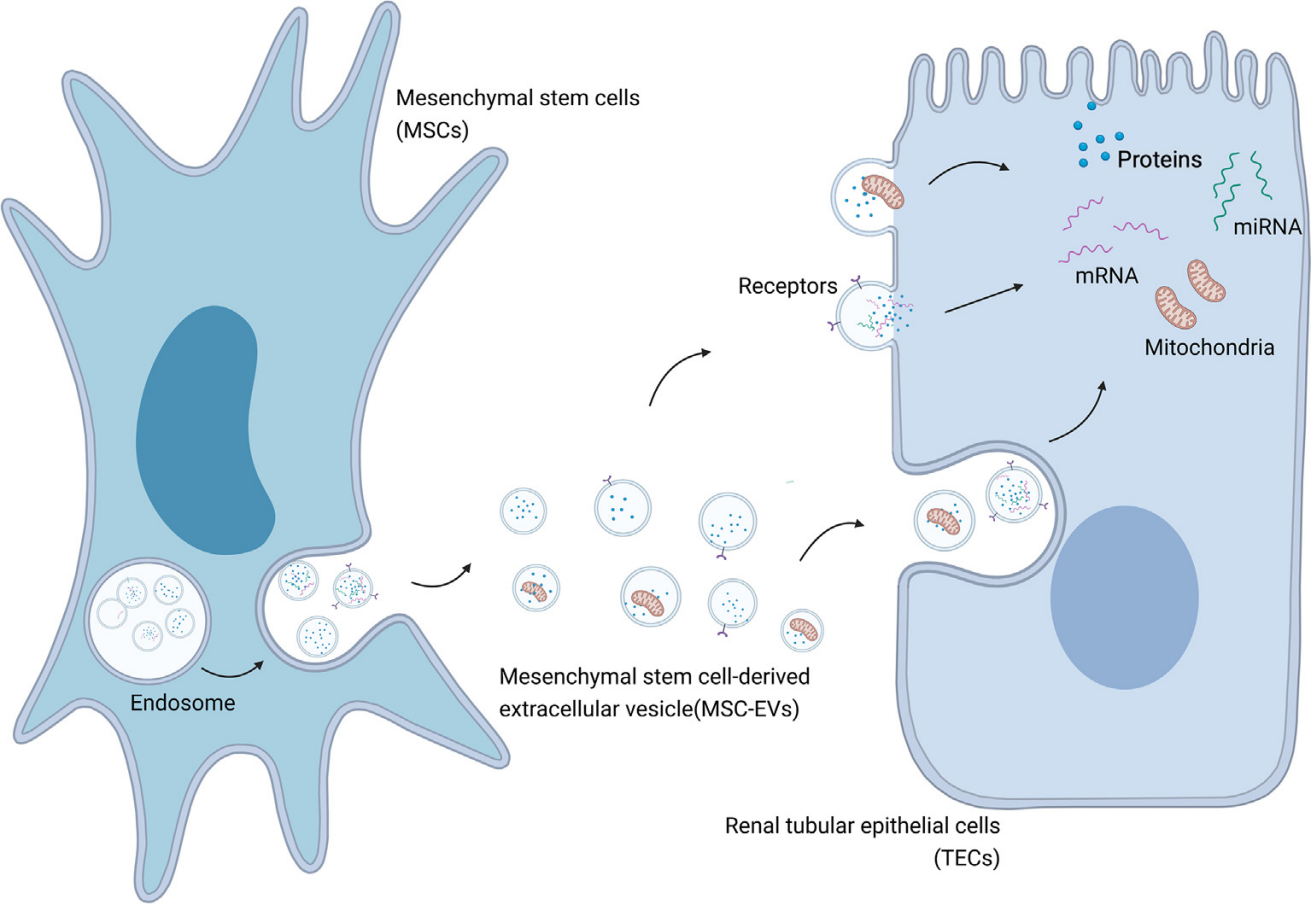
MSC-EVs mediate transportation of biological modules to injured cells in AKI. Created with BioRender.com.

**Figure 2:**
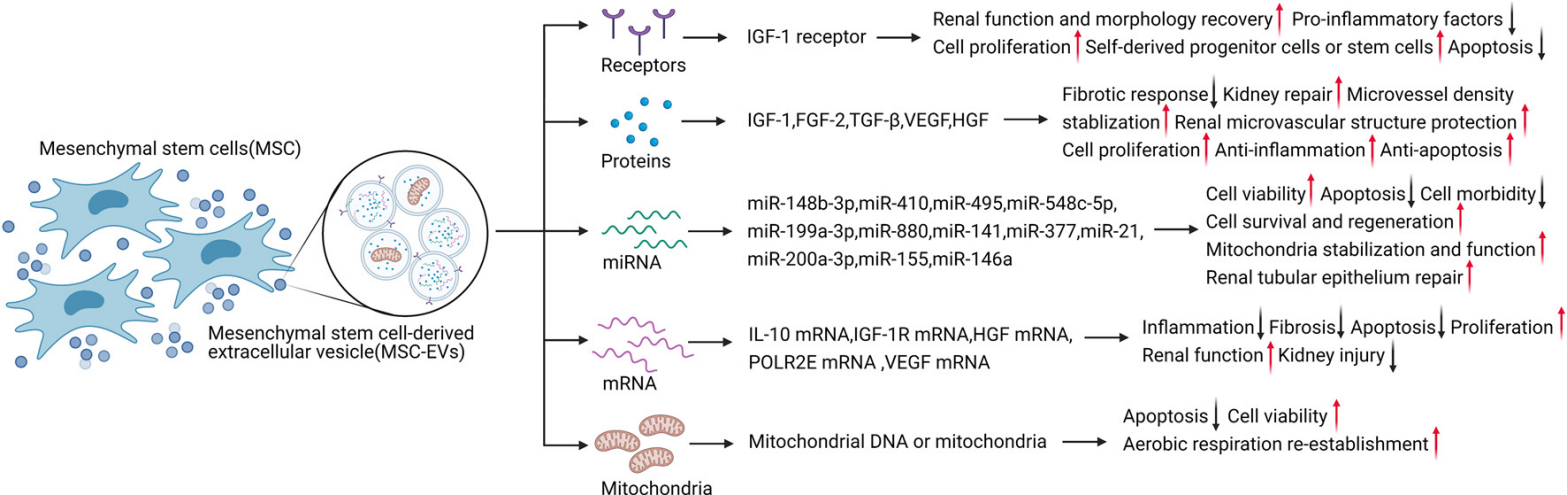
MSC-EVs carry various contents to exert therapeutic effects in AKI. Created with BioRender.com.

Further research into MSC therapy of AKI has shown the presence of MSC-EVs in AKI models treated with MSCs (44). MSCs can also release proteins (43), RNA (45), and mitochondria (46) into injured kidney tissue *via* EVs, in addition to their secretory function (30) (**Figure 1**). Various cytokines and their mRNAs are found in MSC-EVs, and there is gradual recognition that the therapeutic effects of MSCs are mainly mediated by MSC-EVs (43, 47). The effects exerted by MSC-EVs are similar to those reported following MSC administration in previous studies (48). EVs derived from cells such as MSCs act as messengers mediating cell-to-cell communication by carrying a train of biologically active molecules, which is regarded as a critical mechanism in AKI (24, 45). As intercellular messengers, EVs implement the therapeutic effects of MSCs, including regulating the damaged local microenvironment, regulating gene expression in injured kidney cells, improving the survival of damaged cells, resisting apoptosis, regulating inflammation, and reducing mitochondrial damage (49). In MSC-EV-based therapy of AKI, EVs can locate to the injured kidney tissue spontaneously after administration (50). Importantly, this effect is specific to MSC-EVs, involving the EV adhesion molecules CD44 and CD29, while EVs obtained from fibroblasts are ineffective (51). EVs, as endocytic vesicles, are bound to the membrane and are released by eukaryotic cells in an evolutionarily conserved manner, enabling cell-to-cell communication (52). MSC-EVs are generated by the paracrine and secretory functions of MSCs, carrying proteins, lipids, and nucleic acids into injured tissues (53). EVs can also deliver mRNAs and microRNAs (miRNAs) *via* endocytosis to regulate target cells at the transcriptional level (**Figure 2**) (45). As an intercellular messenger, EVs are responsible for regulating the damaged local microenvironment, regulating gene expression in AKI cells, improving the survival of damaged cells, resisting apoptosis and inflammation, and reducing mitochondrial damage (11, 12, 54).

To identify the candidate therapeutic factors in EVs, Eirin et al. used proteomics to characterize the protein content of MSC-EVs derived from porcine MSCs from abdominal fat (42). The expression characteristics of MSC and EV markers detected by liquid chromatography-tandem mass spectrometry proteomics analysis showed that proteins enriched in MSC-EVs were related to a wide range of biological functions, including angiogenesis, blood coagulation, apoptosis, extracellular matrix remodeling, and inflammation regulation (43). Thus, EVs include selectively enriched protein cargo with specific biological characteristics, and these proteins are used for cell-to-cell communication to promote tissue repair. For example, MSC-derived EVs directly transferred IGF-1 receptors to renal tubular cells (13), accelerating kidney repair after AKI and reducing AKI by enhancing the activation of the Nrf2/ARE pathway to exert antioxidant effects (46, 55). Human adipose-derived MSCs (AD-MSCs) activated SOX9 in renal tubules and prevented the transformation of TGF-β1 into the pro-fibrotic phenotype induced by EV shuttling, resulting in anti-fibrotic effects (35, 56, 57).

Cytokines delivered by MSC-EVs and paracrine factors such as HGF, TGF, IGF-1, VEGF, and FGF-2 can regulate inflammation, resist apoptosis, promote damaged cell repair, and prevent fibrosis in AKI (36).

MSC-EV-based treatment of AKI involves the transport of various cytokines, including IGF-1. MSC-EVs secrete IGF-1 receptor mRNA directly to renal TECs, as well as directly secreting IGF-1 and carrying IGF-1 receptors to promote kidney repair in AKI (13). Several studies have indicated that this occurs *via* IGF-1-induced activation of the Akt survival pathway (38). AKI is associated with increased expression of the pro-apoptotic protein Bcl-2-associated X protein (Bax) and decreased expression of anti-apoptotic Bcl-2 (58). EVs can be integrated into injured renal TECs to promote the recovery of renal function and morphology. The beneficial effect of MSCs on renal tissue was related to the inhibition of renal tubular oxidative damage in a cisplatin-induced AKI model, manifested by the expression of nitrotyrosine and induction of Akt phosphorylation (59). IGF-1 activates Akt, as a kinase located on the phosphoinositide-3 kinase (PI3K) signaling pathway with an important regulatory role, through phosphorylation modification to inhibit Bax to resist apoptosis and up-regulate the Bcl-2 survival pathway, and to block mitochondrial caspase-9 to reduce inflammation and resist apoptosis (60). At the same time, IGF-1 activates the MAPK pathway to promote cell proliferation (13, 61). Morigi et al. confirmed that MSC treatment significantly increased phospho-Akt and activated downstream targets for cell proliferation and cell survival in a cisplatin-induced AKI model (56). In an *in vitro* model, proinflammatory factors such as TNF-α and IL-1b significantly reduced proximal tubular cell injury following treatment with MSCs and cisplatin (56). IGF-1 has demonstrated several biological effects in mouse kidney tissues treated with bone marrow MSCs, including promoting cell proliferation, changing the hemodynamics, and reducing apoptosis (13). In the same experimental model, Imberti et al. tested the effects of cord blood MSCs, which can improve kidney function and enhance the protective effect in AKI animal models. As a mitogenic and pro-survival factor, IGF-1 helps recruit self-derived progenitor cells or stem cells, thus, promoting the regeneration process in the kidney (62).

In addition to IGF-1, other growth factors transported by MSC-EVs also protect the microenvironment of the injured site and damaged cells. FGF-2 affects AKI *via* its anti-fibrotic effect, and knockout of FGF-2 blocked the repair process and induced a fibrotic response (63). TGF-β binds to the TGF receptor and activates downstream Smad and non-Smad pathways to perform a variety of biological effects in AKI (64). Acting *via* TGF-β-dependent macrophages, it can prevent ischemic kidney injury and tubular interstitial fibrosis (56, 60, 65). Up-regulation of VEGF receptor-2 can also be observed in kidney tissue during the ischemic AKI response, suggesting that VEGF may also be a potential therapeutic target (66–68). VEGF stabilized microvessel density to protect the renal microvascular structure and promote renal recovery through mitogenic and anti-apoptotic mechanisms (32). HGF can reduce kidney damage by promoting cell proliferation, anti-inflammation, and anti-apoptosis. HGF also promoted kidney repair and the proliferation of kidney cells *via* tyrosine phosphorylation of the c-met receptor in kidney injury induced by IRI and glycerol (13). Moreover, HGF gene therapy and HGF-modified MSCs had a significant therapeutic effect in AKI in an anti-apoptotic manner (13, 69, 70).

MSC-EVs can be internalized into renal TECs to treat AKI at the translational level. Recent studies showed that mRNAs for factors such as IL-10, IGF-1R, HGF, DNA-directed RNA polymerases I, II, and III subunit RPABC1, and VEGF could be loaded into EVs and transported to the target cells to exert translational effects, including anti-inflammation, anti-fibrosis, and anti-apoptosis effects, promoting proliferation, improving renal function, and reducing kidney injury (43, 71). Notably, the efficacy of MSC-EVs was partly mediated by miRNAs loaded in EVs, which directly regulated transcription and translation (50). EV-mediated miRNAs have also been shown to play a significant role in AKI (72). For example, miR-148b-3p and miR-548c-5p promoted cell viability (73, 74), miR-199a-3p reduced AKI *via* anti-apoptotic effects (75), miR-30 stabilized mitochondria, improved renal function, and exerted anti-apoptotic effects (76), and miRNA let-7a-5p reduced cell morbidity and improved cell survival (77, 78). Other miRNAs are regulated and actively participate in the regeneration process involving miR-21 in MSC-EV therapy of AKI, possibly related to renal tubular epithelium repair and internal cell reprogramming (79). EVs can also stabilize mitochondria through miRNAs, especially miRNA-200a-3p, which shuttles to TECs *via* MSC-EVs and targets the Keap1-Nrf2 signaling pathway to normalize mitochondrial membrane potential by reducing mitochondrial fragmentation, increasing the number of mitochondrial DNA copies, and protecting mitochondrial function in TECs during kidney repair (55, 71).

Mitochondria are considered to play an integral role in AKI development (80), suggesting the possibility that mitochondria might be transferred horizontally into kidney cells to reprogram cell metabolism and promote kidney recovery. Mitochondrial DNA or mitochondria can be transported directly into the damaged site by EVs, thus reducing kidney damage (81). Spees et al. first showed in 2006 that MSCs could serve as mitochondrial donors in cell survival (82). Actively transferring healthy mitochondria from MSCs can restore aerobic metabolism and protect cells from being eliminated (83). Plotnikov et al. subsequently confirmed the transport of mitochondria from MSCs to renal tubular cells in normal *in vitro* culture medium (84). In addition to renal tubular cells, vascular endothelial cells are also damaged during AKI. The delivery of mitochondria from MSC-EVs reduced apoptosis and increased cell viability, and restored the normal balance between aerobic respiration and glycolysis, indicating the re-establishment of aerobic respiration (12, 81). EVs derived from MSCs have previously demonstrated huge therapeutic potential in AKI *via* transporting proteins and RNAs with biological activity, and by conveying mitochondria and their DNA directly, suggesting that modifying the contents of MSC-EVs to affect specific signaling pathways may represent a promising therapeutic approach (28, 85).

## EVs Participate in the Immune Regulation of MSCs in AKI

**Figure 3:**
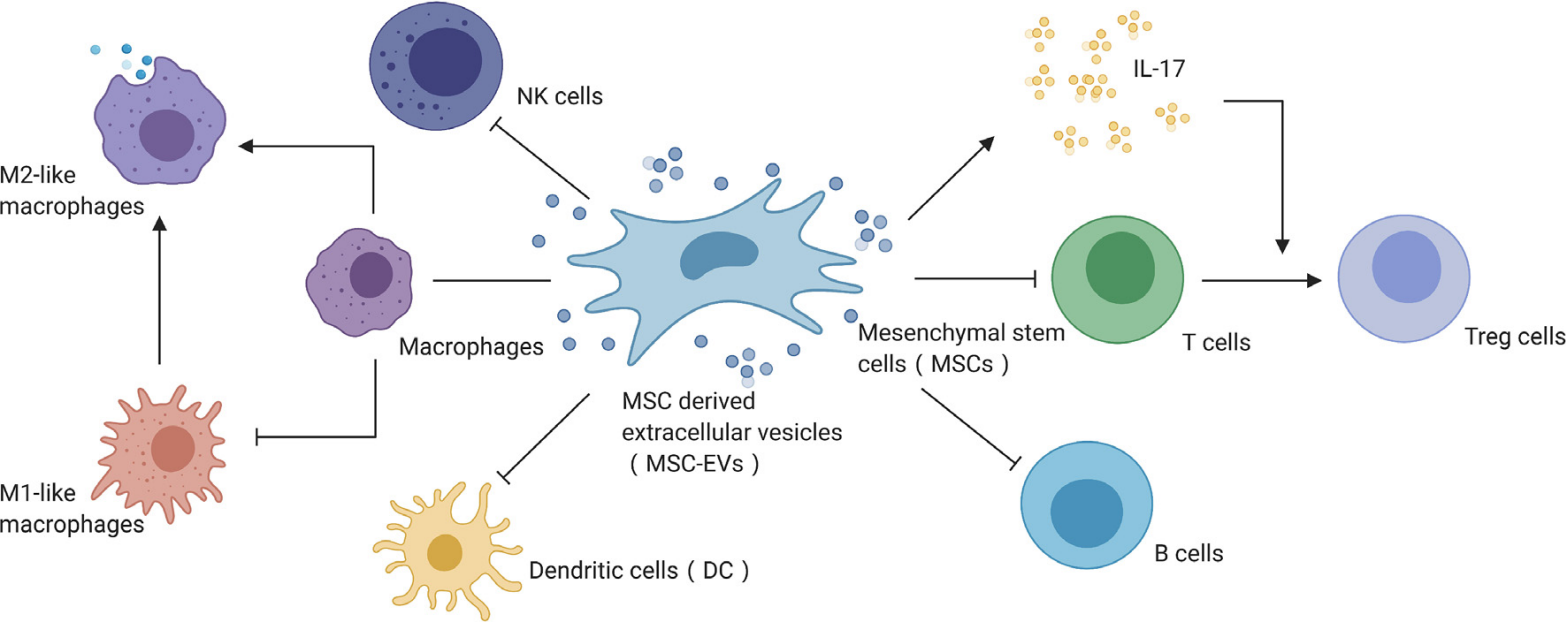
MSC-EVs mediate immune regulation in AKI. Created with BioRender.com.

MSCs-EVs have shown strong immune regulation in AKI treatment (**Figure 3**) and have demonstrated significant regulatory effects in a variety of immune cells, including inhibiting the transendothelial migration and chemotaxis of neutrophils, promoting macrophage M2 type polarization, inhibiting T cell activation, and inhibiting IFN-γ secretion (86–90). These processes were mainly mediated by TNF-α-stimulated gene 6 (TSG-6), which regulates inflammation with multiple functions (91–93). Apart from TSG-6, MSC-EVs also depend on IL-6, IL-10, prostaglandin E2, HGF, and indoleamine2,3-dioxygenase to regulate the immune microenvironment (94, 95), secreting miRNAs involving miR-155 regulate inflammation on the extracellular environment interact with dendritic cells to regulate endotoxin-induced inflammation (96–99). In addition, MSC-derived signals mediated by EVs can inhibit the proliferation of natural killer cells, reduce the activity of B lymphocytes, and secrete IL-17 to promote T cell transformation into Treg cells (86, 100).

As noted above, AKI is usually associated with disorders and abnormal activation of the immune system, which can in turn be affected by treatment with MSCs and their EVs, suggesting that utilizing and modifying EVs to regulate the immunomicroenvironment might be an efficient and effective therapeutic approach for AKI (100–106). Although some researchers have reported that MSCs and MSC-EVs can affect the immune function and immune microenvironment in AKI both *in vitro* and *in vivo*, the findings have been inconsistent (107). Unfortunately, the specific mechanism by which MSC-EVs act as immune mediators remains elusive, and further research into the therapeutic mechanisms of EVs in AKI is warranted.

## MSC-EVs Are Superior to MSCs and Can Be Modified Artificially as Medication Carriers, With Rare Adverse Reactions

Although many preclinical studies have shown the effectiveness of MSCs and MSC-EVs in AKI, few clinical trials have utilized MSC-EVs for the clinical treatment of patients (49, 108, 109). Compared with MSCs, MSC-EVs generate a reduced inflammatory response with lower immunogenicity after administration, with beneficial effects in terms of administration dose and frequency, as well as reducing stem cell-associated risks such as cytokine release syndrome, ectopic tissue caused by poor differentiation, and tumorigenesis (31, 48, 110–113). In a comparative study, MSC-EVs were at least as effective as MSCs, indicating that many of the therapeutic effects of MSCs could be attributed to EVs (114, 115). In addition, MSCs may have more efficient homing ability than EVs (116, 117).

Preclinical studies *in vitro* and *in vivo* showed that EVs may have advantages over MSCs and may thus have great potential for future stem cell therapies (118, 119). First, MSC-EVs are highly stable and suitable for long-term storage without the need to add potentially toxic cryopreservatives (120, 121). Second, MSC-EVs can transfer functional proteins and miRNAs directly to recipient cells, inducing stronger signal transmission *via* cell-to-cell communication (45). Third, MSC-EVs have a lower risk of a rejection reaction, and an immune response after allogeneic application is rare (72). In addition, MSC-EVs avoid the potential tumorigenicity of MSCs, with no evidence of carcinogenic potential to date and no signs of unwanted differentiation (24, 28, 110). In contrast, MSCs have the possibility of tumorigenesis and poor differentiation (122). One study reported that MSC-EVs with anti-tumor activity inhibited tumor growth both *in vivo* and *in vitro* (123).

EVs have important unique characteristics. MSC-EVs may have a weaker homing ability than MSCs, which could partly reduce the accuracy of EV-based therapy; however, EVs are safe, with few adverse reactions (24, 28). EVs also show plasticity, and their contents can be modified artificially to improve and enhance not only their homing ability, but also their therapeutic effects (28, 29, 85). However, robust evidence is still lacking, and more research involving animal models and clinical studies is needed.

## How to Administer MSC-EVs

When administered *via* peripheral intravenous injection, most MSCs or MSC-EVs distribute to the lung, spleen, or celiac lymph nodes, thus, reducing their therapeutic efficacy (36). Moreover, the homing ability of EVs is lower than that of MSCs. However, a recent study showed that renal artery administration could transport more EVs and generate better therapeutic effects to injured kidney tissue with greater precision compared with other administration routes (30, 50, 124). Renal artery puncture in clinical patients may be performed under ultrasound image guidance (124). However, although EV injection *via* the renal artery provides a possible approach, this administration route is more difficult and is associated with ethical concerns in clinical practice (49, 114).

Bruno et al. utilized MSC-derived microvesicles, a kind of EVs, in lethal cisplatin-induced AKI and showed that increased administration times improved the therapeutic effects due to anti-apoptosis in AKI. The single administration of EVs ameliorated renal function and morphology and improved survival, but had no effect on chronic tubular injury and persistent increases in blood urea nitrogen (BUN) and creatinine. They also found that using multiple injections of EVs significantly reduced the mortality of mice, and mice surviving at day 21 showed normal histology and renal function (125).

Recent meta-analyses investigating the effects of administration and cell source on the therapeutic effects of EVs have indicated the importance of these factors in clinical research and applications. A meta-analysis using serum creatinine (Scr) as an indicator of efficacy compared the timing of administration in various studies (between 1 h and 3 days after the occurrence of AKI), and showed a better treatment effect following administration of MSC-EVs within 1 h after the occurrence of AKI, suggesting that they should be administered as early as possible (114). Current research mainly focuses on EVs secreted by MSCs derived from adipose tissue, bone marrow, and cord blood. However, the source tissue also has an impact on MSC-EVs (47, 126). For example, compared with cord blood-derived MSCs, signals mediated by EVs derived from bone marrow MSCs had greater effects on bone growth and differentiation (49, 114). In addition, adipose-derived MSCs had similar immune regulation effects to bone marrow-derived MSCs (17). The EV source should be selected flexibly according to the type of kidney injury and treatment needs. Meanwhile, because MSC from different sources have different characteristics, the EVs secreted by them will also differ. The therapeutic effects of EVs from other sources of MSCs in AKI are still unclear, and there is much need for further research. Bone marrow-derived MSCs may be more likely to express specific proteins *via* lentiviral expression vector transduction, such as angiogenin-1, IGF-1, and Akt, thus, influencing the restructuring and repair of injured tissue (13). This research could indicate the ability to utilize lentiviral vectors to modify MSCs to produce EVs with specific efficacies (127).

## How to Improve the Therapeutic Effect of MSC-EVs

Several studies have focused on improving MSC-EVs in a variety of areas (128) (**Table 1**). These studies have mainly involved improving the technology for the isolation of EVs, investigating potential administration routes, the use of pulsed focused ultrasound (pFUS), preconditioning EVs, and inducing the overexpression of EVs by lentiviral vector transduction.

**Table 1:** Recent research into the therapeutic effects of MSC-EVs.

Authors	Title	Year	EVs source	AKI model	Intervention	Effects
Lee, JH et al. (128)	Reproducible large-scale isolation of exosomes from adipose tissue-derived mesenchymal stem/stromal cells and their application in acute kidney injury	2020	Adipose tissue-derived MSCs	Cisplatin-induced AKI	Produce ASC-EVs with tangential flow filtration	EV yield↑; EV quality↑
Cao, J et al. (127)	Three-dimensional culture of MSCs produces exosomes with improved yield and enhanced therapeutic efficacy for cisplatin-induced acute kidney injury	2020	Fresh human umbilical cord-derived MSCs	Cisplatin-induced AKI	Produce MSC-EVs with a hollow fiber bioreactor-based three-dimensional culture system	EV yield↑; EV quality↑; therapeutic efficacy↑; collection efficiency↑; efficiency of TECs uptake↑
Ullah, M et al. (129)	Reversing acute kidney injury using pulsed focused ultrasound and MSC therapy: a role for HSP-mediated PI3K/AKT signaling	2020	Bone marrow-derived MSCs	Cisplatin-induced AKI	Combine pFUS pretreatment of the kidney with MSC-derived EVs	No significant improvement in homing ability of EVs; kidney injury markers↓; renal function↓; inflammation↓; apoptosis↓; cell proliferation↑
Ullah, M et al. (130)	HSP70-mediated NLRP3 inflammasome suppression underlies reversal of acute kidney injury following extracellular vesicle and focused ultrasound combination therapy	2020	Bone marrow-derived MSCs	Cisplatin-induced AKI	Combine pFUS pretreatment of the kidney with MSC-derived EVs	HSP70↓; NLRP3 inflammasome↓;IL-1↓; IL-18↓; therapeutic effects of MSC-EVs↑; anti-inflammation↑; cell regeneration↑
Ullah, M et al. (131)	Pulsed focused ultrasound enhances the therapeutic effect of mesenchymal stromal cell-derived extracellular vesicles in acute kidney injury	2020	Bone marrow-derived MSCs	Cisplatin-induced AKI	Combine pFUS pretreatment of the kidney with MSC-derived EVs	MAPK/ERK↑; PI3K/Akt↑; eNOS↑; SIRT3↑; kidney injury markers↓; renal function↓; inflammation↓; apoptosis↓; cell proliferation↑; survival↑
Zhang, C et al. (77)	Supramolecular nanofibers containing arginine-glycine-aspartate (RGD) peptides boost therapeutic efficacy of extracellular vesicles in kidney repair	2020	Human placenta-derived MSCs	Ischemic reperfusion injury-induced AKI	Precondition EVs with RGD peptides	Stability and retention of MSC-EVs↑; anti-fibrosis in the chronic phase↑; kidney injury↓; cell proliferation↑; EV integrin-mediated loading↑
Liu, Y et al. (132)	Enhanced therapeutic effects of MSC-derived extracellular vesicles with an injectable collagen matrix for experimental acute kidney injury treatment	2020	Human placenta-derived MSCs	Ischemic reperfusion injury-induced AKI	Precondition EVs with collagen matrix	Angiogenesis↑; apoptosis↓; stability and retention of MSC-EVs↑; therapeutic efficacy↑
Alzahrani, FA et al. (133)	Melatonin improves therapeutic potential of mesenchymal stem cells-derived exosomes against renal ischemia-reperfusion injury in rats	2019	Bone marrow-derived MSCs	Ischemic reperfusion injury-induced AKI	Precondition EVs with melatonin	Kidney damage↓; inflammation; renal regeneration↑; angiogenesis↑; anti-oxidation↑; oxidative stress↓
Zhang, ZY et al. (134)	Oct-4 enhanced the therapeutic effects of mesenchymal stem cell-derived extracellular vesicles in acute kidney injury	2020	Human umbilical cord-derived MSCs	Ischemic reperfusion injury-induced AKI	Overexpress Oct-4 by lentiviral vector transduction	Apoptosis↓; Scr↓; BUN↓; renal fibrosis↓; renal tubular epithelial cell proliferation↑

EV, extracellular vesicle; MSC, mesenchymal stem cell; AKI, acute kidney injury; pFUS, pulsed focused ultrasound; HSP70, heat shock protein 70; NLRP3, NLR Family, Pyrin Domain Containing Protein 3; IL, interleukin; TEC, tubular epithelial cell; RGD, arginine-glycine-aspartate; MAPK, mitogen-activated protein kinase; ERK, extracellular regulated protein kinase; Scr, serum creatinine; BUN, blood urea nitrogen; eNOS, endothelial nitric oxide synthase.

EVs are traditionally separated by ultracentrifugation, but this method has major limitations, including the co-precipitation of EVs with contaminants including protein aggregates, loss of EV function due to aggregation or distortion during the isolation process, and functional inhibition of EVs by the media used for density gradient ultracentrifugation (129). AKI is common in clinical practice, and improved large-scale production of EVs would be needed to satisfy the requirements if EVs were applied for the clinical treatment of these patients (1). Large-scale isolation of single-batch EVs by ultracentrifugation is also restricted by the limited instrument capacity (130). Lee et al. showed that it was possible to isolate adipose tissue-derived MSC-EVs stably and reproducibly on a large scale without loss of function using tangential flow filtration (131), with successful life-preserving effects in a cisplatin-induced lethal rat model of AKI. Other studies have indicated that the production of EVs by three-dimensional (3D) culture of MSCs could improve the efficacy and increase yield. Cao et al. produced EVs in 3D culture and showed that, compared with conventional 2D culture, the 3D culture system increased the total yield of EVs 19.4-fold, thus, indicating that the 3D culture system had a higher EV-collection efficiency. Surprisingly, EVs obtained by 3D culture were taken up more efficiently by renal TECs, showing better anti-inflammatory effects and increasing the survival rate of TECs *in vitro* (130).

pFUS was shown to alter the kidney microenvironment to enhance homing of subsequently infused MSCs. Mujib et al. investigated if the combined use of pFUS with MSC-EVs could improve the therapeutic effect by improving the homing ability of EVs in AKI (132). Surprisingly, although pFUS did not up-regulate local cytokine expression or improve bone marrow MSC homing, it did enhance the therapeutic efficacy of MSC-EVs in AKI. Further analysis showed that this effect was related to the up-regulation of heat shock protein (HSP) 20/HSP40 and subsequent PI3K/Akt signaling. This indicated that pFUS had independent as well as synergistic therapeutic effects in the context of AKI, and is thus, a promising affiliated method for MSC-EV therapy (132). Further studies of the combined use of MSC-EVs and pFUS showed that pFUS affected HSP70-mediated NLRP3 inflammasome suppression to improve the anti-inflammatory and regenerative effects (133). In addition, Mujib et al. also showed that pFUS upregulated the proliferative signaling (MAPK/extracellular signal-regulated kinase, PI3K/Akt) and regenerative pathways (endothelial nitric oxide synthase, sirtuin 3) to suppress inflammation in AKI (134).

The low stability and retention of MSC-EVs have partly limited their therapeutic efficacy, and methods involving novel biological materials such as arginine-glycine-aspartate (RGD) peptides and collagen matrix have been investigated with the aim of improving these features. Zhang et al. developed an RGD peptide scaffold to increase EV integrin-mediated loading, and found that RGD hydrogels facilitated MSC-EVs containing miRNA let-7a-5p, which improved their repair potential in AKI (78). Liu et al. isolated EVs from human placental mesenchymal stem cells and wrapped them in a collagen matrix, and *Gaussia* luciferase imaging confirmed that the collagen matrix effectively encapsulated the EVs and augmented their efficacy in AKI by improving the stability and promoting the sustained release of EVs (135). Biological modules can be used to cover EVs and interact with the microenvironment in AKI to improve the positive effects of MSC-EV-based therapy (78, 135, 136). However, few studies have investigated the mechanisms involved in the interaction between the injured tissue microenvironment and biological modules covering EVs. This warrants further investigation to guide clinical trials aimed at identifying the most efficient artificial biological modules to improve the therapeutic effects of MSC-EVs.

Scientists have also tried to improve the effects of MSC-EVs in AKI by preconditioning them with medications to prevent further damage deterioration and help renal recovery. Alzahrani et al. tested MSC-EVs preconditioned with melatonin in AKI induced by IRI with bilateral renal artery clipping, and showed that melatonin-preconditioned MSC-EVs performed better than untreated EVs in terms of alleviating kidney damage and inflammation, promoting regeneration, angiogenesis, and anti-oxidation, and inhibiting oxidative stress (136). EVs can also be artificially modified to overexpress specific modules. Zhang et al. transduced MSCs with Oct-4 *via* a lentiviral vector to produce Oct-4-overexpressing MSCs, which significantly reduced attenuated apoptosis, Scr and BUN levels, promoted renal TEC proliferation, and rescued renal fibrosis in IRI-induced AKI (137).

Recent studies concentrating on improving the limitations of MSC-EV-based therapy showed that it was possible to safely modify MSC-EVs and enhance their therapeutic effects. As noted above, their homing ability and tissue stability limit the therapeutic effects of EVs, but these issues can be overcome by the use of collagen matrix and RGD peptides (78, 135). In addition to enhancing the effects of EVs, 3D culture and tangential flow filtration may also allow the large-scale clinical application of EVs (130, 131). However, MSC-EVs still face numerous challenges and limitations before they can be clinically applied in patients.

## Limitations and Future Perspectives of MSC-EVs

Various challenges still need to be overcome before MSC-EVs can be utilized in clinical treatments. First, there is significant heterogeneity between MSC-EVs in terms of size, leading to variations in the secreted components and functional characteristics of the EVs (15). Further studies are therefore needed to choose the specific size of EVs according to their cargo (138). Second, the isolation and storage methods of EVs may affect their therapeutic efficacy (139). Ensuring the quality of EVs is an important problem, and producers are supposed to confirm that the EVs are derived from cellular matrix, rather than being components from cells damaged during cryopreservation and mechanical failure (130, 131, 135). It is advisable to optimize and confirm the preservation conditions to maintain the efficacy of the EVs after defrosting (128). Importantly, the follow-up time in previous studies was only between 1 day and 2 weeks, which is inadequate for evaluating the long-term outcome and prognosis (114), and more clinical trials with long-term follow-up are therefore needed to provide more robust evidence. Catering for the demands of large-scale clinical applications and producing enough high-quality MSCs then become critical issues (99). In addition, even though EVs have demonstrated similar effects to MSCs, their homing ability is much weaker than that of MSCs, representing a limitation of MSC-EV-based therapy that needs improving. Zhang et al. recently used monocyte mimics to enhance the homing ability of MSC-EVs to the injured site in a myocardial IRI model, suggesting a possible approach for improving the homing ability of MSC-EVs for the therapy of AKI (140). Regarding monitoring the distribution of EVs, their physicochemical properties may be affected by some lipophilic markers, which could affect the observations (13), and new tracing markers will be needed to detect the distribution and effects of EVs in clinical practice. Increasing research is currently focused on investigating ways of modifying the cargo of MSC-EVs to improve their therapeutic efficiency (28, 141). Clinical trials are currently required to verify and approve the use of customized EVs and to assess the safety and tolerance of modified MSC-EVs.

## Conclusion

MSC-EVs are responsible for the main therapeutic effects of MSCs in AKI, and demonstrate specific therapeutic effects and improve the efficacy of regenerative stem cell therapies. However, the lack of clinical trials means that MSC-EVs still face many challenges before they can be used for clinical treatment. Nonetheless, we believe that MSC-EVs will become an effective approach to overcome the current limitations of AKI treatment.

## Author Contributions

J-KL and CY drafted the manuscript. ZL, G-WT and YS conceived the proposal, revised the manuscript and provided funding support. J-CL, M-HL and D-LH collected literatures and revised the manuscript. ZL and G-WT helped the language editing and provided funding support. All authors contributed to the article and approved the submitted version.

## Funding

This article was supported by grants from the Research Funds of Shanghai Municipal Health Commission (2019ZB0105), Natural Science Foundation of Shanghai (20ZR1411100), Program of Shanghai Academic/Technology Research Leader (20XD1421000), National Natural Science Foundation of China (82070085), Clinical Research Funds of Zhongshan Hospital (2020ZSLC38 and 2020ZSLC27), and Smart Medical Care of Zhongshan Hospital (2020ZHZS01).

## Conflict of Interest

The authors declare that the research was conducted in the absence of any commercial or financial relationships that could be construed as a potential conflict of interest.

The handling editor has declared past co-authorships with one of the authors (CY) in the time of review.
